# Prehospital Evaluation of Effusion, Pneumothorax, and Standstill (PEEPS): Point-of-care Ultrasound in Emergency Medical Services

**DOI:** 10.5811/westjem.2015.5.25414

**Published:** 2015-07-14

**Authors:** Sundeep R. Bhat, David A. Johnson, Jessica E. Pierog, Brita E. Zaia, Sarah R. Williams, Laleh Gharahbaghian

**Affiliations:** * Stanford University School of Medicine, Department of Emergency Medicine, Stanford, California; † Kaiser Permanente Santa Clara Medical Center, Department of Emergency Medicine, Santa Clara, California; ‡ Emergency Medicine Physicians, Department of Emergency Medicine, Mecklenberg, North Carolina; § Kaiser Permanente San Francisco Medical Center, Department of Emergency Medicine, San Francisco, California

## Abstract

**Introduction:**

In the United States, there are limited studies regarding use of prehospital ultrasound (US) by emergency medical service (EMS) providers. Field diagnosis of life-threatening conditions using US could be of great utility. This study assesses the ability of EMS providers and students to accurately interpret heart and lung US images.

**Methods:**

We tested certified emergency medical technicians (EMT-B) and paramedics (EMT-P) as well as EMT-B and EMT-P students enrolled in prehospital training programs within two California counties. Participants completed a pre-test of sonographic imaging of normal findings and three pathologic findings: pericardial effusion, pneumothorax, and cardiac standstill. A focused one-hour lecture on emergency US imaging followed. Post-tests were given to all EMS providers immediately following the lecture and to a subgroup one week later.

**Results:**

We enrolled 57 prehospital providers (19 EMT-B students, 16 EMT-P students, 18 certified EMT-B, and 4 certified EMT-P). The mean pre-test score was 65.2%±12.7% with mean immediate post-test score of 91.1%±7.9% (95% CI [22%–30%], p<0.001). Scores significantly improved for all three pathologic findings. Nineteen subjects took the one-week post-test. Their mean score remained significantly higher: pre-test 65.8%±10.7%; immediate post-test 90.5%±7.0% (95% CI [19%–31%], p<0.001), one-week post-test 93.1%±8.3% (95% CI [21%–34%], p<0.001).

**Conclusion:**

Using a small sample of EMS providers and students, this study shows the potential feasibility for educating prehospital providers to accurately identify images of pericardial effusion, pneumothorax, and cardiac standstill after a focused lecture.

## INTRODUCTION

The use of bedside point-of-care ultrasound (US) in the emergency department (ED) has been increasing over the past two decades, and is now routinely used by emergency physicians as part of the diagnostic workup of sick patients and screening of trauma victims. It has decreased the time to life-saving interventions for many conditions. For example, use of the extended-focused assessment with sonography for trauma (E-FAST) exam by emergency physicians accurately identifies fluid in the abdomen requiring urgent blood transfusion or exploratory laparotomy, pericardial effusion requiring immediate evacuation, or pneumothorax requiring immediate decompression.^1^–^5^ It is now considered standard-of-care in advanced trauma life support.^6^

Emergency medical service (EMS) providers have the opportunity to diagnose, initiate treatment, and stabilize life-threatening conditions within the first critical minutes of a patient’s decompensation. US has been used by physicians, flight nurses, and EMTs, on both ground and air ambulance teams in several countries in Europe^7^ as well as by emergency physicians in military combat.^8^ Several international studies have shown prehospital bedside US can be conducted with accurate interpretation by physician and non-physician providers, allowing specific interventions to be performed or hospital preparations to be made.^9^–^12^ These studies were of emergency or prehospital physicians, or trained sonographers.^8^,^11^–^12^ To date, there is limited literature on the use of prehospital US in the United States.

Prior studies have demonstrated that flight medics and ground EMS providers can obtain and interpret images for abdominal aortic aneurysm assessment, FAST exam screening, and cardiothoracic US images.^13^–^16^ A recent case report demonstrated that prehospital emergency US allowed paramedics to accurately identify a clinically significant pericardial effusion in a stabbing victim, allowing them to report this to the trauma surgeon prior to arrival.^17^ A recent 2013 study (the PAUSE pilot) examined professional paramedics’ ability to acquire and interpret images using a protocol to diagnose pneumothorax, pericardial effusion, or cardiac standstill, finding that after a 2-hour didactic program the providers had an accurate recognition score of 9.1 out of 10. However, this single-center study was limited to 20 trained paramedics.^18^ A separate study found that aeromedical prehospital personnel at a Level I trauma center had significant improvement in scores on both a written exam and observed clinical examination after undergoing a structured, 2 month training curriculum. However, these providers were critical care paramedics and nurses who already had significant clinical knowledge, and the study focused primarily on the E-FAST modality.^19^ In addition, many of the studies, including the PAUSE pilot, also demonstrate adequate image acquisition ability of prehospital providers,^9^,^14^,^18^ and that these images are not subject to inaccuracy even when obtained in moving transport vehicles.^12^,^16^

There remain significant gaps and limitations in existing studies regarding the ability of prehospital providers to acquire and interpret point-of-care US images. Here, we aimed to determine if EMS providers would be able to 1) accurately identify the presence or absence of pericardial effusion, pneumothorax, and cardiac standstill after a one-hour didactic course, and 2) retain the ability to interpret the images over time.

## METHODS

We conducted a prospective, observational study of certified emergency medical technicians (EMT-B) and EMT-paramedics (EMT-P) as well as students enrolled in prehospital training programs within two counties in California. The institutional review board approved the study. Participants were recruited from four EMS training programs, and study sessions were held at each of these training programs with written consent obtained from participants.

Inclusion criteria for the study were age greater than or equal to 18 years, enrollment in an EMS training course and/or current certification as an EMS provider, and ability to attend all sessions (pre-testing, lecture, and post-testing) during the study. Subjects were excluded if they were below the age of 18, were not involved within the county EMS system as either an actively enrolled student or certified EMS provider, did not consent to participation in the study, and/or were unable to attend the required sessions.

Study sessions were held prior to or after scheduled classes for the local prehospital training programs; certified EMS providers were also invited to attend these study sessions. Study participants were first asked to complete an anonymous demographics questionnaire including gender, age, educational status, EMS affiliation, and prior US experience. This was followed by a multiple-choice question (MCQ) pre-test that included 16 full-motion and still US clips of normal and abnormal pathology. They then received a one-hour didactic lecture covering basic scanning technique, normal US anatomy, and image interpretation of both normal and pathologic heart and lung imaging videos. This included presence or absence of pericardial effusion, pneumothorax, and cardiac standstill. Immediately following the lecture, study participants were given a post-test consisting of the same video clips in different order with different questions from the pre-test. For one of the local prehospital training classes, the same post-test was administered one-week later. The test contained 16 image-questions, with six of the images having been shown in the lecture and 10 novel images. The images were originally obtained in the ED setting by emergency physicians with prior US training and knowledge. While the repeat post-test did contain the same questions from the immediate post-test, subjects were not given answers or feedback on their initial tests. Participants were asked to self-rate their confidence level with US interpretation and given one minute to answer each MCQ on both the pre- and post-tests. Scores were determined as percentage of questions answered correctly on the test. Subjects did not acquire any of the US images.

Both the pre- and post-tests were validated using a population of emergency medicine physicians (both attendings and residents) knowledgeable on bedside point-of-care US, but who had no prior knowledge of the test images. The validation tests were administered to the physician group without receipt of the lecture intervention and prior to utilization of the tests for the study participants. All data were analyzed using two-tailed, paired t-tests in SPSS 11.0 (Chicago, IL).

## RESULTS

We enrolled 57 prehospital providers (49 male, mean age and SD 26.2 years±7.0) consisting of 19 EMT-B students, 16 EMT-P students, 18 certified EMT-B providers, and 4 certified EMT-paramedics (Table 1). There was no prior US experience in 84% of subjects. Of those who reported prior US experience, this consisted primarily of observing emergency providers conducting US scans during the EMS providers’ shadowing shifts in the ED. Test images were validated by 11 emergency physicians with a pre-test score of 98.9% and post-test score of 99.4% (95% CI [−18%–70%], p=0.34).

**Figure 1:**
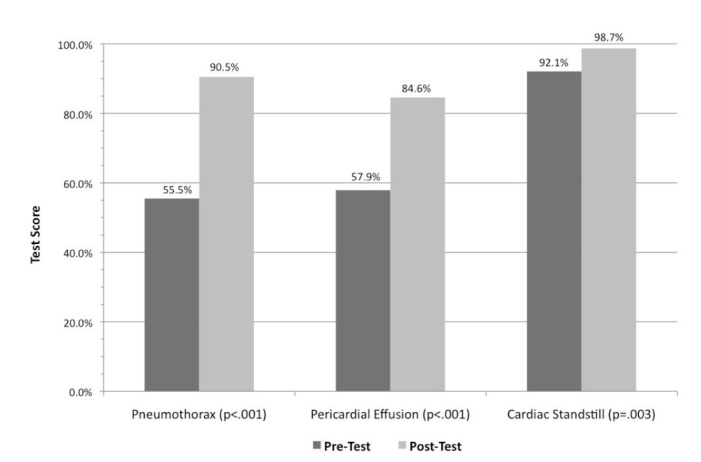
Pre- and post-test scores among all certified pre-hospital providers and students significantly improved (p<0.05) among each modality after a focused one-hour didactic lecture.

There was a significant improvement for all subjects between the pre- and post-tests with a mean pre-test score of 65.2%±12.7% and a mean immediate post-test score of 91.1%±7.9% (95% CI [22%–30%], p<0.001). Scores significantly improved for all three individual pathologies as shown in Figure 1. The mean pre-test overall score for cardiac standstill was 92.1%±15.1% with a mean immediate post-test score increase to 98.6%±5.6% (95% CI [11%–23%], p=0.003). The mean score for pericardial effusion improved from 57.9%±26.3% pre-test to 84.6%±21.5% immediate post-test (95% CI [35%–19%], p<0.001) and the mean score for pneumothorax increased from 55.5%±20.9% pre-test to 90.6%±9.82% (95% CI [29%–41%], p<0.001) immediate post-test.

Among the certified EMS providers (N=22), scores showed significant increases in mean score pre-test (63.9%±16.6%) to immediate post-test (93.4%±6.5%, 95% CI [22%–37%], p<0.001). Among these providers, scores for identification of pneumothorax and pericardial effusion showed significant increases after subjects received the focused lecture. There was no significant change for identification of cardiac standstill: pre-test score 90.9%±18.1% and post-test score 98.9%±5.3% (CI [−17%–6.9%], p=0.069) (Table 2).

The repeat post-test was administered one week later to 19 EMT-B students. Post-test scores remained significantly higher than pre-test scores (Table 3). There was no significant difference between the immediate and repeat post-test mean scores.

**Figure 2:**
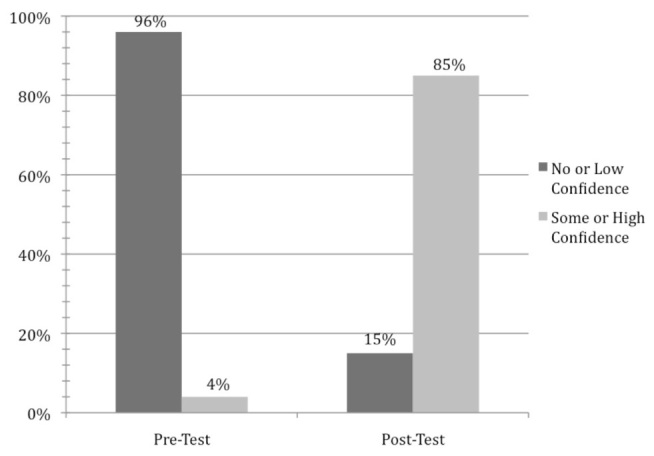
Study participants reported markedly higher confidence in their ultrasound interpretation skills after a focused, one-hour didactic lecture. N-value for pre-test is 55 subjects, and N-value post-test of 54 subjects.

Among all subjects, self-reported confidence with point-of-care US increased after the study intervention. During the pre-test, 53 participants (96%) reported no or low confidence with US interpretation. During the immediate-post test, only 8 participants (15%) reported no or low confidence whereas 46 subjects (85%) reported some or high confidence with US interpretation (Figure 2).

## DISCUSSION

Although use of bedside ultrasonography within United States EDs is now common, the use of this technology in the field by EMS providers is limited. Use of bedside ultrasonography in the prehospital setting has the potential to provide EMS providers with important diagnostic data and assist with difficult treatment and transport decisions in the field. Furthermore, as EMS systems within the United States evolve to focus on transport to specialty centers, such as trauma or cardiac care centers, prehospital US could play a role in guiding these decisions, just as they have in Europe.^15^,^20^ To date, data regarding the ability of EMT-B and EMT-P providers to accurately interpret US imaging is limited, with only a few pilot studies having examined this question. Our results demonstrate that prehospital providers are able to gain the ability to interpret US images for specific life-threatening pathologies after a brief and focused lecture. This study adds to the growing body of literature demonstrating that EMS providers within the United States can accurately interpret point-of-care bedside US images.

Our study findings concur with previous reports supporting the feasibility of formal US instruction and subsequent accurate US interpretation by EMS providers. Previous studies, however, were primarily conducted among international EMS subjects,^9^–^10^,^15^,^21^ military providers,^13^ or with subjects having prior clinical experience and training in US.^11^,^14^,^19^,^22^ Our study supports the findings of the previously discussed PAUSE protocol^18^ but builds on it by including a larger cohort of prehospital providers, and incorporating both certified prehospital paramedics and EMTs, as well as EMT students. Further, we showed that after the focused lecture, providers could not only interpret US images accurately, but also reported increases in their confidence with US interpretation and retained the knowledge over time.

Knowledge retention among the cohort of EMT-B students was high one week after the teaching session, and this effect was seen across all three pathologies tested. This complements prior work that showed retention of pneumothorax identification by prehospital providers in a cadaveric model up to nine months after initial teaching by prehospital providers.^22^ A recent study showed that aeromedical providers were able to successfully demonstrate image acquisition and accurate interpretation of E-FAST imaging after 6 weeks; however, their study was based on a time-intensive training curriculum among providers who already had significant clinical experience.^19^

Our results suggest that incorporation of a succinct and relevant one-hour didactic on prehospital point-of-care US into the EMS training curriculum could have a large and lasting impact on the providers’ skill set, even at a junior level, without much burden on already stretched curricula. In addition, the didactic material can be focused in scope, emphasizing those emergencies for which early identification might change treatment or triage decisions. Given a growing body of literature regarding potential impacts of prehospital identification of pneumothorax, pericardial effusion or cardiac standstill, we chose to focus our one-hour curriculum on these three modalities.

Our findings validate European studies on pneumothorax evaluation, which demonstrated prehospital providers’ ability to correctly identify lung sliding.^9^,^11^,^15^–^16^ Several studies have outlined needle thoracostomy failure in the prehospital setting.^23^–^25^ Evaluation of the presence or absence of pneumothorax could obviate unnecessary thoracostomy or indicate need for a repeat attempt after failed thoracostomy in the field. Similarly, presence or absence of lung sliding post-intubation by EMS providers could be used to confirm appropriate endotracheal tube placement.^22^,^26^–^27^

Presence of cardiac standstill during non-traumatic arrest in the field could affect the need for transport to the ED given the growing body of evidence that patients with cardiac standstill have a nearly zero percent chance of survival to hospital discharge.^28^–^29^ Interestingly, this modality may be the most feasible and intuitive to teach EMS providers based on our results. Mean scores for this modality were greater than 90% pre-test and for all-comers, significantly improved to approach 99% on immediate post-testing. This suggests that EMS providers may be able to identify cardiac standstill with enough precision that it could assist their ability to declare patients deceased in the field or assess need for transport to an acute care facility. In fact, in a study of Dutch physicians working in a prehospital helicopter system, in nine of 60 patients (15%) the physician made a decision to stop all prehospital treatment and resuscitation, based on cardiac US in the field.^15^

Our results also provide validation that EMS providers can identify the presence of pericardial effusion after receiving brief instruction. The significance of this modality in the prehospital setting is especially relevant among penetrating thoracic trauma patients, in whom early identification of an effusion or tamponade may expedite thoracotomy or pericardiocentesis upon ED arrival.^17^,^30^ However, this modality may prove challenging when signs are subtle. In our cohort, although scores significantly increased among all participants, the mean post-test score remained just under 85% accurate, compared with greater than 90% accuracy on the other two modalities. Thus, the clinical impact of this potentially more challenging study and potential false negative findings in the field may need to be explored further.

## LIMITATIONS

Our study represents the evaluation of prehospital providers’ ability to interpret US images. The results do show that these providers can acquire and retain US interpretation skills and confidence. Limitations include the fact that this was an observational trial using a convenience sample of EMS volunteers, with limited sample sizes. It is possible that these volunteers may not represent the rank-and-file EMS student. Demographics and confidence data were acquired by self-report, which could be skewed by bias. However, this would not affect the objective statistical improvement in scores after our study intervention. Because participation was voluntary and did require attendance at both the didactic and post-testing sessions for inclusion in the retention cohort, it is possible that the retention cohort contained selection-bias from individuals more enthusiastic about US thus affecting results with a bias toward improvement. Additionally, this cohort had a smaller sample size due to our ability to have a follow-up session one week later with only one EMS class, thus potentially impacting statistical significant and generalizability of results. Similarly, our specific analysis of certified providers should be taken in the context that the majority of these providers were EMT-B trained with very few EMT-P trained individuals. While the analysis combined all certified providers into one cohort, the statistical significance of our findings could be skewed by the small sample of EMT-P individuals, and further, it would be difficult to analyze the results as to whether US may be more easily taught to either EMT-B or EMT-P certified individuals.

Our study utilized test images that were obtained within the ED setting and although they were validated prior to use, the images may represent idealized versions of findings when compared with those that are obtained by EMS providers themselves or subject to other environmental factors while obtaining US in the prehospital setting. Though this may limit the strength of our findings, several prior studies have shown that EMS providers can obtain adequate images,^9^,^14^,^18^ and that these images are not affected by moving transport vehicles.^12^,^16^ There may be some bias introduced through utilizing the same images on post-testing and repeat-testing one week later; however, providers were not given directed feedback nor access to the testing materials within the interim period, thus we feel this is only a small limitation on the validity of our results.

Our study demonstrates the potential ability of EMS students and certified providers to acquire and retain knowledge of US interpretation with regard to specific pathologies. However, the impact on patient care and transport remains to be determined. Given studies noting the changes in management and transport to appropriate levels of care by European providers, the same may hold true for EMS US use and decision-making in the United States.^15^,^20^

There remains a tangible cost to implementation of US in the EMS field, with the need to obtain and maintain equipment. Although our chosen modalities – pneumothorax, pericardial effusion, and cardiac standstill – could likely have potential benefits for triage, transport, and treatment of prehospital patients, the true impact and cost-effectiveness of such decisions has yet to be determined. Ongoing studies will need to assess prehospital providers’ acquisition of US, and potential delays in treatment or transport within EMS systems in the United States. There may also be differences in utility and clinical impact within urban versus rural communities. Future study could also focus on longitudinal tracking of individual EMS providers to evaluate the number of USs being completed, image acquisition ability, and also longitudinal knowledge retention and skills.

## CONCLUSION

This study showed potential promise for training prehospital EMS providers in accurate US interpretation through a one-hour didactic lecture focused on US technique and anatomy for the assessment of pericardial effusion, pneumothorax, and cardiac standstill. Using a small group of EMS students and providers, subjects’ performance on US image interpretation increased from 60% to 90% after training and was maintained at one week. Additionally, subjects reported increased confidence in their comfort level with US interpretation. Although limited, our findings lend potential support to existing data that demonstrate prehospital providers may be able to sufficiently gain and retain knowledge of point-of-care US interpretation for pericardial effusion, pneumothorax, and cardiac standstill.

## Figures and Tables

**Table 1:** Participant characteristics (n=57).

Demographics	Number of participants
Male gender	49 (86.0%)
Mean age ± SD (years)§	26.24±7.03
Emergency medical service affiliation	
EMT student	19 (33.3%)
Paramedic student	16 (28.1%)
Certified EMT	18 (31.6%)
Certified paramedic	4 (7.0%)
Highest level of education completedψ	
High school	38 (66.7%)
Undergraduate	14 (24.6%)
Master’s	4 (7.0%)
Prior ultrasound experience†	
Formal education	2 (3.6%)
Informal training	7 (12.7%)
None	46 (83.6%)

*EMT*, emergency medical technician

§ N=54

ψ N=56

† N=55

**Table 2:** Scores for certified pre-hospital providers (n=22).

	Pre-test	Immediate post-test	p-value (95% CI)
Total score	63.9±16.7	93.5±6.5	p<0.001 (22%–37%)
Pneumothorax	52.8±24	92.6±10	p<0.001 (28%–52%)
Pericardial effusion	59.1±34.1	89.7±14.8	p<0.001 (17%–45%)
Cardiac standstill	90.9±18.2	98.9±5.3	p=0.069 (−17%–6.9%)

Scores reported as mean(%) ± SD(%), p-values are calculated using two-tailed, paired t-test.

**Table 3:** Scores for repeat post-testing among emergency medical technicians students (n=19).

	Pre-test	Immediate post-test	1-week post-test	Pre- vs. 1-week p-value (95% CI)	Immediate vs. 1-week p-value (95% CI)
Total score	65.8±10.7	90.5±7.0	93.1±8.3	p<0.001 (21%–34%)	p=0.134 (−6.1%–8.9%)
Pneumothorax	55.3±21	91.4±9.4	95.4±10.4	p<0.001 (30%–51%)	p=0.083 (−8.4%–5.6%)
Pericardial effusion	61.8±21	80.3±22.9	82.9±20.5	p=0.004 (7.6%–35%)	p=0.706 (−17%–12%)
Cardiac standstill	90.8±12.4	98.7±5.7	98.7±5.7	p=0.03 (8.8%–15%)	p=1.0 (−4.0%–4.0%)

Scores reported as mean(%) ± SD(%), p-values are calculated using two-tailed, paired t-test.
